# Hemodynamic responses detected by exercise echocardiography in patients with ankylosing spondylitis and psoriatic arthritis

**DOI:** 10.3389/fmed.2025.1688233

**Published:** 2026-01-12

**Authors:** Szilárd Burcsár, Blanka Morvai-Illés, Albert Varga, Attila Balog, Gergely Ágoston

**Affiliations:** 1 Department of Rheumatology and Immunology, Faculty of Medicine, Albert Szent-Györgyi Health Centre, University of Szeged, Szeged, Hungary; 2 Department of Family Medicine, Faculty of Medicine, Albert Szent-Györgyi Health Centre, University of Szeged, Szeged, Hungary

**Keywords:** ankylosing spondylitis, psoriatic arthritis, stress echocardiography, cardiopulmonary manifestations, chest wall abnormalities

## Abstract

**Introduction:**

Cardiopulmonary complications are common in ankylosing spondylitis (AS) and psoriatic arthritis (PsA) and have an adverse impact on the mortality and quality of life of patients. Myocardial involvement can lead to systolic and diastolic dysfunction, which can be asymptomatic for a long time. Chest wall rigidity, a complication of both diseases, can also lead to cardiac dysfunction, especially in the right heart-pulmonary circulation unit. We aimed to assess the response of the pulmonary circulation-right ventricular function unit and left ventricular function during exercise in AS and PsA patients and hypothesized that stress echocardiography may unmask the early non-invasive hemodynamic changes caused by chest wall rigidity and left heart involvement.

**Methods:**

A total of 72 participants were enrolled in the study: 26 had AS, 18 had PsA, and 28 healthy individuals were matched by age and sex. To assess the maximally tolerated workload, all subjects underwent resting and exercise stress echocardiography on a supine bicycle ergometer. Echocardiographic measurements were taken at rest, at 50 watts workload, and at maximal exercise. Detailed clinical characteristics were also assessed, including the advanced ankylotic axial status of both patient groups.

**Results:**

At rest, only pulmonary vascular resistance (PVR) values were significantly higher in patients with AS and PsA than in controls. During exercise, the tricuspid regurgitation velocity (TRV) was significantly increased in the AS and PsA groups. PVR stress was significantly higher in patients with AS and PsA than in controls. *E*/*e*′, which refers to estimation of the left ventricular filling pressure, significantly increases during stress in patients with AS and in the ankylotic group compared to controls at peak stress. In patients with AS and those with PsA, the disease duration was strongly correlated with *E*/*e*′ mean measured during peak stress but not with TRV or PVR.

**Discussion:**

Stress echocardiography is a promising method for assessing subclinical cardiopulmonary changes among AS and PsA patients. Changes in PVR during stress may highlight pulmonary complications related to chest wall restriction and remodeling of the pulmonary vasculature at the subclinical stage.

## Introduction

Spondyloarthropathies (SpA) include a group of overlapping chronic inflammatory rheumatic diseases. Ankylosing spondylitis (AS) and psoriatic arthritis (PsA) are the two most frequent diseases in the SpA group and share several similar clinical features. The most common manifestations of AS are chronic back pain and spinal stiffness. Psoriasis represents the most common extra-articular manifestation in patients with PsA. The primarily involved joints include the sacroiliac joints, axial skeleton, peripheral joints, tendons, and the entheses in SpA. Following peripheral joints, tendons and entheses involvement are frequent not only in AS but also in PsA patients. In addition to joint complications, a higher prevalence of cardiovascular (CVD) comorbidities was demonstrated in AS and PsA patients, with a worse disease outcome (1). CVD is one of the most common comorbidities in AS, following osteoporosis, spinal fractures, and fibromyalgia (2, 3). Traditional CVD risk factors such as hypertension, obesity, diabetes, and the metabolic syndrome are prevalent in PsA patients (3). Along with these traditional risk factors, chronic inflammation associated with atherosclerosis, pharmacological treatments such as NSAIDs and corticosteroids, and other CV risk factors such as alcohol, tobacco, and a sedentary lifestyle, also contribute to chronic disabling diseases (4–6). Patients with PsA also have cardiovascular and pulmonary manifestations, such as valvular abnormalities, conduction disturbances, and, rarely, pulmonary fibrosis (7). CVD is the leading cause of death among PsA with patients (8, 9). Patients with PsA have a 68, 22, and 31% increased risk of myocardial infarction (MI), cerebrovascular disease, and heart failure, respectively, compared with the general population (10). In AS, multiple cardiovascular structures can be affected. The most frequent manifestation is aortitis, leading to aortic insufficiency and conduction disturbances. Although aortic insufficiency has been reported in nearly 30% of AS patients, clinical manifestations occur less frequently (1–10%). Conduction disturbances such as atrioventricular blocks, intraventricular blocks, and bundle-branch blocks have been detected regularly (3–33%) due to inflammation and fibrosis of the interventricular septum (4). Patients with AS are also more likely to have disrupted ventricular depolarization compared to healthy controls. Pericarditis, mitral valve abnormalities, and myocardial involvement may also occur in AS, especially left ventricular (LV) systolic and diastolic dysfunction (11). Ischemic heart disease is also a more frequent manifestation in patients with AS and PsA compared to the general population (12). There is also evidence that patients with AS without cardiovascular risk factors have a higher prevalence of left ventricular systolic and diastolic dysfunction compared to the healthy control group. AS has also been suggested as an independent risk factor for atrial fibrillation, particularly in younger male patients (<40 years) (13, 14).

Axial involvement in AS and PsA is observed with many similarities and only a few differences between the two diseases (15). The advanced outcome of axial involvement is ankylosis, both in AS and PsA. Chest wall rigidity is related to the involvement of the costovertebral, costosternal, and thoracic vertebral joints with their fusions. It can lead to respiratory abnormalities, including the most common manifestations, such as restrictive ventilatory changes. On the other hand, restrictive ventilatory changes resulting from the ankylosis of the chest wall and left ventricular diastolic dysfunction due to the involvement of the myocardium do not always cause symptoms or complaints in the early stages and may present in a subclinical form or can complicate other acute lung diseases. Abnormal pathophysiological changes in these restrictive lung defects can only manifest during exercise or can complicate other acute lung diseases (16). Semi-supine exercise stress echocardiography is a promising method for detecting subclinical hemodynamic abnormalities (14). Due to the physiologic nature of physical exercise, multiple changes in the cardiac function and right ventricular-pulmonary circulation unit, as well as variations in blood pressure, alterations in the electrocardiogram, and exercise tolerance, can be assessed by echocardiography. It is considered the safest type of stress echocardiography, which is another advantage of this imaging modality (17, 18). The potential effect of axial involvement and chest wall rigidity on pulmonary circulation and myocardial function in AS and PsA patients remain insufficiently investigated.

For this purpose, we conducted a study to assess the pulmonary circulation and the right and left ventricular function in AS patients and PsA patients with or without advanced ankylosis before and during semi-supine exercise stress echocardiography.

## Patients and methods

### Study population

In our case–control study, 72 participants were enrolled in our cardiology and rheumatology outpatient clinics. Twenty-six patients with AS and 18 patients with PsA were diagnosed based on the Assessment of Spondyloarthritis International Society (ASAS) classification criteria for AS (19, 20) and Classification Criteria for Psoriatic Arthritis (CASPAR) for PsA (21), respectively. All 44 AS and PsA patients had axial involvement, and 21 out of 44 patients had ankylosis (18 AS and 3 PsA patients). Twenty AS and PsA patients had peripheral arthritis (Tables 1–3). The advanced structural changes in the spine and sacroiliac joints were diagnosed by X-ray in the ankylosing group. Twenty-eight age- and sex-matched healthy controls were compared to the patients’ group.

**Table 1:** Shows echocardiographic parameters and differences at rest and peak stress in patients with ankylosing spondylitis (AS) and a matched control group.

Parameter	AS patients (*n* = 26)	Control group (*n* = 26)	Significance
Age (years)	51.38 ± 11.61	54.08 ± 10.23	0.38
Sex (male %)	22 (84.61%)	22 (84.61%)	
Heart rate rest (/min)	79.77 ± 12.98	72.62 ± 13.84	0.06
Heart rate stress (/min)	128.15 ± 17.51	132.58 ± 17.89	0.37
Systolic RR rest (mmHg)	121.77 ± 20.05	124.00 ± 18.39	0.68
Systolic RR stress (mmHg)	171.77 ± 29.40	192 ± 21.14	0.006
Diastolic RR rest (mmHg)	83 ± 11.77	81.09 ± 10.89	0.56
Diastolic RR stress (mmHg)	90.27 ± 13.44	92.88 ± 9.84	0.43
Maximal workload (watt)	122.12 ± 32.6	140.38 ± 36.7	0.06
LVEF rest (%)	63.64 ± 7.6	67.22 ± 5.5	0.07
LVEF stress (%)	76.00 ± 11.33	68.89 ± 13.49	0.46
LAVI (mL/m^2^)	27.86 ± 8.91	29.93 ± 9.32	0.60
Cardiac index at rest (mL/min/m^2^)	2.35 ± 0.6	2.28 ± 0.8	0.16
Cardiac index at stress (mL/min/m^2^)	5.51 ± 1.49	5.56 ± 1.54	0.10
Septal *e*′ at rest	8.27 ± 2.25	9.46 ± 2.54	0.42
Septal *e*′ at stress	11.96 ± 3.43	13.96 ± 3.84	0.74
Lateral *e*′ at rest	11.5 ± 4.01	12.54 ± 3.37	0.54
Lateral *e*′ at stress	**15.00 ± 4.12**	**16.96 ± 3.54**	**0.002**
*E*/*e*′ mean at rest	6.75 ± 1.68	6.38 ± 1.57	0.07
*E*/*e*′ mean at stress	8.05 ± 2.53	7.57 ± 1.63	0.52
TRV at rest (m/s)	1.77 ± 0.51	1.64 ± 0.43	0.08
TRV at stress (m/s)	**2.74 ± 0.71**	**2.19 ± 0.48**	**0.02**
PASP at rest (mmHg)	23.53 ± 7.37	20.48 ± 6.41	0.25
PASP at stress (mmHg)	31.98 ± 15.95	29.69 ± 8.21	0.53
PVR rest (Wood unit)	**1.85 ± 1.25**	**0.98 ± 0.63**	**0.006**
PVR stress (Wood unit)	**1.79 ± 1.05**	**0.98 ± 0.61**	**0.002**
TAPSE rest (mm)	26.81 ± 4.32	27.54 ± 4.24	0.34
TAPSE stress (mm)	**29.42 ± 5.76**	**28.92 ± 4.79**	**0.01**
Coupling rest (mm/Hgmm)	1.24 ± 0.42	1.55 ± 0.71	0.13
Coupling stress (mm/Hgmm)	1.18 ± 0.6	1.08 ± 0.32	0.55

LVEF, left ventricular ejection fraction; LAVI, left atrial volume index; TRV, tricuspid regurgitation velocity; PASP, pulmonary arterial systolic pressure; PVR, pulmonary vascular resistance; TAPSE, tricuspid annular peak systolic excursion.

Estimation of left ventricular filling pressure: dividing the early mitral inflow velocity (E wave) by the early diastolic mitral annular velocity (e’ wave).

**Table 2:** Shows echocardiographic parameters and differences at rest and peak stress in patients with psoriatic arthritis (PsA) and a matched control group.

Parameters	PsA patients (*n* = 18)	Control group (*n* = 18)	Significance
Age (years)	51.17 ± 13.59	50.17 ± 11.86	0.815
Sex (male%)	14 (77.78%)	14 (77.78%)	
Heart rate rest (/min)	71.39 ± 7.85	72.83 ± 16.02	0.734
Heart rate stress (/min)	128.50 ± 20.62	130.94 ± 17.22	0.702
Systolic RR rest (mmHg)	124.87 ± 19.82	121.53 ± 15.95	0.596
Systolic RR stress (mmHg)	180.72 ± 21.84	185.00 ± 24.75	0.591
Diastolic RR rest (mmHg)	84.35 ± 11.34	81.47 ± 7.48	0.388
Diastolic RR stress (mmHg)	95.61 ± 29.12	89.41 ± 8.39	0.404
Maximal workload (watt)	131.94 ± 32.99	150.00 ± 33.21	0.111
LVEF rest (%)	67.39 ± 8.41	67.29 ± 5.93	0.97
LVEF stress (%)	73.80 ± 5.68	72.50 ± 12.21	0.729
LAVI (mL/m^2^)	26.15 ± 10.07	30.37 ± 10.66	0.237
Cardiac index at rest (mL/min/m^2^)	2.11 ± 0.54	2.46 ± 0.66	0.091
Cardiac index at stress (mL/min/m^2^)	5.42 ± 1.38	5.59 ± 1.57	0.735
Septal *e*′ at rest	8.94 ± 2.70	9.22 ± 2.92	0.77
Septal *e*′ at stress	12.11 ± 3.69	13.22 ± 3.92	0.387
Lateral *e*′ at rest	11.53 ± 3.50	11.94 ± 4.25	0.755
Lateral *e*′ at stress	16.06 ± 4.50	17.06 ± 5.01	0.433
*E*/*e*′ mean at rest	7.45 ± 2.48	7.05 ± 2.02	0.433
*E*/*e*′ mean at stress	8.28 ± 2.49	7.68 ± 2.03	0.433
TRV at rest (m/s)	1.67 ± 0.55	1.61 ± 0.43	0.714
TRV at stress	2.22 ± 0.67	2.15 ± 0.41	0.73
PASP at rest (mmHg)	21.01 ± 6.97	19.86 ± 6.54	0.62
PASP at stress (mmHg)	21.33 ± 12.41	28.13 ± 8.63	0.65
PVR rest (Wood unit)	1.06 ± 0.87	0.80 ± 0.65	0.331
PVR stress (Wood unit)	1.02 ± 0.79	0.93 ± 0.58	0.706
TAPSE rest (mm)	25.56 ± 4.17	27.28 ± 4.36	0.234
TAPSE stress (mm)	27.89 ± 6.04	29.18 ± 5.16	0.504
Coupling rest (mm/Hgmm)	1.31 ± 0.380	1.51 ± 0.60	0.236
Coupling stress (mm/Hgmm)	**1.77 ± 1.07**	**1.10 ± 0.36**	**0.02**

LVEF, left ventricular ejection fraction; LAVI, left atrial volume index; TRV, tricuspid regurgitation velocity; PASP, pulmonary arterial systolic pressure; PVR, pulmonary vascular resistance; TAPSE, tricuspid annular peak systolic excursion.

Estimation of left ventricular filling pressure: dividing the early mitral inflow velocity (E wave) by the early diastolic mitral annular velocity (e’ wave).

**Table 3:** Shows echocardiographic parameters and differences at rest and peak stress in patients with ankylosis and a matched control group.

Parameters	Ankylosis (*n* = 21)	Control group (*n* = 21)	Significance
Age (years)	54.57 ± 11.13	56.00 ± 10.10	0.665
Sex (male%)	20 (95.24%)	20 (95.24%)	
Heart rate rest (/min)	80.81 ± 13.26	76.00 ± 12.95	0.241
Heart rate stress (/min)	128.48 ± 19.77	130.57 ± 19.09	0.729
Systolic RR rest (mmHg)	125.33 ± 20.06	128.10 ± 15.76	0.627
Systolic RR stress (mmHg)	178.38 ± 29.63	189.95 ± 28.05	0.201
Diastolic RR rest (mmHg)	83.67 ± 12.28	81.85 ± 11.13	0.623
Diastolic RR stress (mmHg)	89.52 ± 12.03	92.71 ± 11.13	0.378
Maximal workload (watt)	125.00 ± 32.60	135.71 ± 39.19	0.341
LVEF rest (%)	**62.10 ± 7.85**	**67.25 ± 6.62**	**0.031**
LVEF stress (%)	73.30 ± 12.94	67.00 ± 14.02	0.337
LAVI (mL/m^2^)	26.98 ± 10.14	31.13 ± 11.32	0.224
Cardiac index at rest (mL/min/m^2^)	2.44 ± 0.62	2.36 ± 0.85	0.749
Cardiac index at stress (mL/min/m^2^)	5.56 ± 1.45	5.35 ± 1.32	0.64
Septal *e*′ at rest	**7.52 ± 1.72**	**8.62 ± 1.75**	**0.047**
Septal *e*′ at stress	**11.05 ± 3.54**	**13.71 ± 3.98**	**0.027**
Lateral *e*′ at rest	**9.81 ± 3.23**	**12.52 ± 3.67**	**0.015**
Lateral *e*′ at stress	**13.52 ± 4.07**	**17.40 ± 4.12**	**0.027**
*E*/*e*′ mean at rest	7.37 ± 1.81	6.34 ± 1.74	0.068
*E*/*e*′ mean at stress	**8.96 ± 3.03**	**7.21 ± 1.65**	**0.027**
TRV at rest (m/s)	1.70 ± 0.50	1.61 ± 0.44	0.597
TRV at stress	**2.65 ± 0.74**	**2.18 ± 0.50**	**0.02**
PASP at rest (mmHg)	22.53 ± 7.33	19.59 ± 6.29	0.138
PASP at stress (mmHg)	30.12 ± 16.81	28.91 ± 9.38	0.775
PVR rest (Wood unit)	**1.59 ± 1.27**	**0.92 ± 0.65**	**0.043**
PVR stress (Wood unit)	**1.74 ± 1.17**	**0.98 ± 0.65**	**0.014**
TAPSE rest (mm)	25.76 ± 4.71	27.43 ± 4.27	0.237
TAPSE stress (mm)	29.71 ± 5.29	29.37 ± 4.72	0.829
Coupling rest (mm/Hgmm)	1.24 ± 0.41	1.63 ± 0.77	0.058
Coupling stress (mm/Hgmm)	1.27 ± 0.62	1.11 ± 0.35	0.307

LVEF, left ventricular ejection fraction; LAVI, left atrial volume index; TRV, tricuspid regurgitation velocity; PASP, pulmonary arterial systolic pressure; PVR, pulmonary vascular resistance; TAPSE, tricuspid annular peak systolic excursion.

Estimation of left ventricular filling pressure: dividing the early mitral inflow velocity (E wave) by the early diastolic mitral annular velocity (e’ wave).

No comorbidities were detected in patients and controls that could have influenced our investigation, including the absence of atrial fibrillation; no prior history of interstitial lung disease, chronic obstructive pulmonary disease, bronchial asthma, or resting pulmonary hypertension (PH); absence of moderate or severe aortic or mitral valve disease on the screening echocardiogram; no history of cardiomyopathies or heart failure; absence of severe kidney failure or anemia (eGFR ≤30 mL/min, Hgb ≤100 g/L); and absence of malignancy. Written informed consent was obtained from all subjects, and our study was reviewed and approved by an independent ethical committee of the university (14/2017-SZTE). The study adhered to the tenets of the most recent revision of the Declaration of Helsinki.

### Ultrasound assessment

A comprehensive transthoracic echocardiogram (TTE) at rest was performed using a Vivid-S70 (GE Vingmed, Horten, Norway) ultrasound machine equipped with a 3S probe (1.5–3.6 MHz). An experienced cardiologist with EACVI-TTE certification performed all measurements according to the recommendations of the American Society of Echocardiography and the European Association of Cardiovascular Imaging (22). Then, stress echocardiography on a semi-recumbent bicycle ergometer was performed according to the recommendations of the European Association of Cardiovascular Imaging/American Society of Echocardiography 15 with an incrementally increasing workload of 25 watts every 2 min (23). All the resting and stress Doppler assessments were performed in a semi-supine position. In addition to the standard parameters, particular attention was paid to the tricuspid regurgitation velocity (TRV) and velocity-time integrals of the left and right ventricular outflow tracts (LVOT VTI and RVOT VTI). Pulmonary vascular resistance (PVR) was calculated from the equation proposed by Roule [PVR = 32.8*(TRV/LVOT VTI)] (24) and expressed in Wood units (WU). To assess the relationship between right ventricular (RV) contractility and pulmonary afterload at rest and during stress, we used pulmonary arterial coupling, defined as the ratio of tricuspid annular plane systolic excursion (TAPSE) and pulmonary artery systolic pressure (PASP) (25). TRV was used to estimate the pressure gradient between the right ventricle and right atrium, forming the basis for the estimation of PASP using the simplified Bernoulli equation PASP = 4*TRV^2^ + (right atrial pressure estimated from the dimensions of the inferior vena cava) (24). A TRV value of 2.8 m/s is established as the cutoff for resting measurements according to the European pulmonary hypertension guidelines (26). The cutoff value for TRV during stress is generally considered 3.5 m/s (27). Pathological thresholds for PVR (>2 WU) and TAPSE/PASP (<0.55 mm/mmHg) are also declared in the guidelines (26). The end-diastolic filling pressure was estimated by *E*/*e*′ mean, which was calculated by dividing the early diastolic mitral inflow velocity (*E*) by the average of early diastolic mitral annular lateral and septal velocities (*e*′). Continuous 12-lead ECG monitoring was performed throughout the examination, and blood pressure was measured at the beginning of every new stage. Pulse oximetry was used to assess capillary oxygen saturation at rest and at peak stress. The cardiologist was blinded to the patient’s rheumatologic diagnoses, including their ankylosis status.

### BASDAI and DAS28 scores

Two guideline-recommended severity scores assess disease activity. The Bath Ankylosing Spondylitis Disease Activity Index (BASDAI) score is defined as a patient-reported outcome measure that assesses disease activity in axial spondyloarthritis (axSpA). It contains a six-question survey, with scores ranging from 0 to 10, where higher scores indicate more severe symptoms. A BASDAI score of ≥4/10 is considered indicative of active disease status (28). Disease Activity Score 28 (DAS28) is not only a measure of disease activity in rheumatoid arthritis but also in peripheral joint involvement in psoriatic arthritis. The DAS228 score combines a clinical assessment of 28 specified peripheral joints, serum inflammatory markers (C-reactive protein or erythrocyte sedimentation rate), and a visual analog scale of the patient’s functional status. The level of disease activity can be interpreted as low (DAS28 ≤3.2), moderate (3.2 <DAS28 ≤5.1), or high (DAS28 >5.1) (29).

### Statistical analysis

Continuous variables with a skewness of −1 to +1 were accepted as normally distributed, but a visual inspection of histograms was also applied. Our data are expressed as numbers and percentages for categorical variables, as mean ± standard deviation for normally distributed continuous variables, and as median with interquartile range for non-normally distributed continuous variables. Univariate comparisons were performed on normally distributed continuous variables using an independent samples *t*-test. The correlations were analyzed using Spearman’s rank correlation for the non-normally distributed data. Adjustments for age were performed using the multivariable linear regression analysis. A *p*-value of <0.05 was accepted as statistically significant. As only a limited number of comparisons were performed, no correction for multiple testing was applied. Data were analyzed using IBM SPSS 22 statistical software.

## Results

All AS and PsA patients had been diagnosed at least 2 years prior to the study. The median disease duration was 13 (5–18) years for AS and 5.5 (3–22.5) years for PsA. BASDAI and DAS28 scores were assessed at the time of the exercise stress echocardiography. The median BASDAI and DAS28 activity scores were 1.70 (1.43–1.90) and 0 (0–1.63) for AS, and 0 (0–0) and 2 (1.33–2.16) for PsA, indicating remission. Patients were treated with biologic DMARDs (91.3%) and conventional DMARDs (34.1%).

We acquired all the resting and stress parameters for patients with AS, patients with PsA, and healthy controls. Further analyses of these parameters were conducted based on patient subgroups, including ankylosis, disease duration, peripheral involvement, and medication.

**Figure 1:**
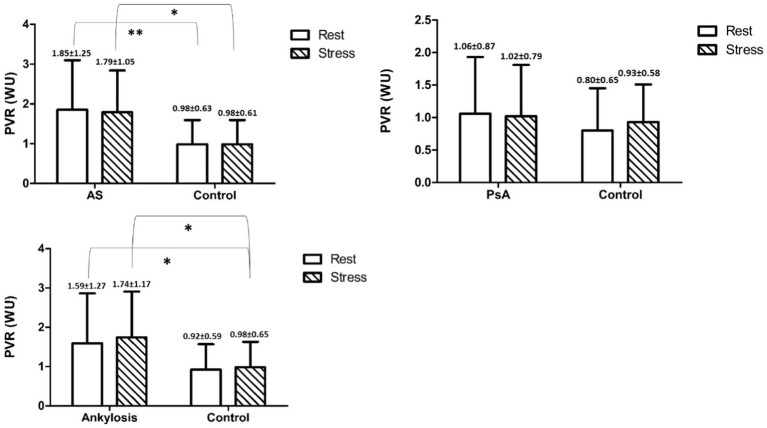
Shows mean PVR values with SD in and between the groups at rest and peak stress. * indicates *p* < 0.05, ** indicates *p* < 0.01.

**Figure 2:**
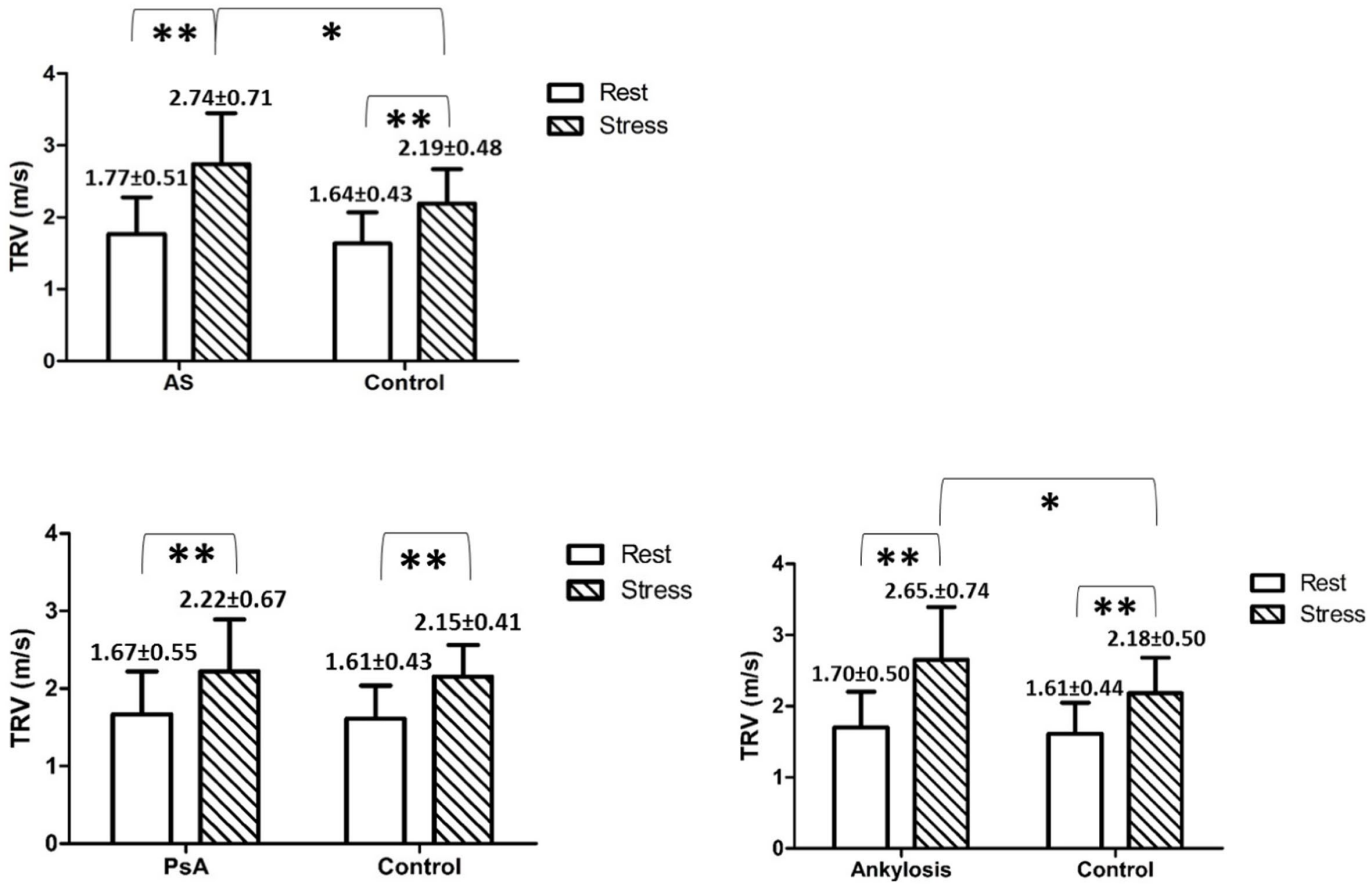
Shows mean TRV values with SD in and between the groups at rest and peak stress. * indicates *p* < 0.05, ** indicates *p* < 0.01.

The PVR values were significantly higher among patients in the AS group and patients with ankylosis, but not among patients in the PsA group, compared with controls, both at rest and under stress (Tables 1–3 and Figures 1, 2).

During exercise, the TRV was significantly increased in all investigated groups compared to rest values. Furthermore, during exercise, the TRV was significantly increased in AS and in patients with ankylosis but not in PsA, compared to controls (Tables 1–3).

**Figure 3:**
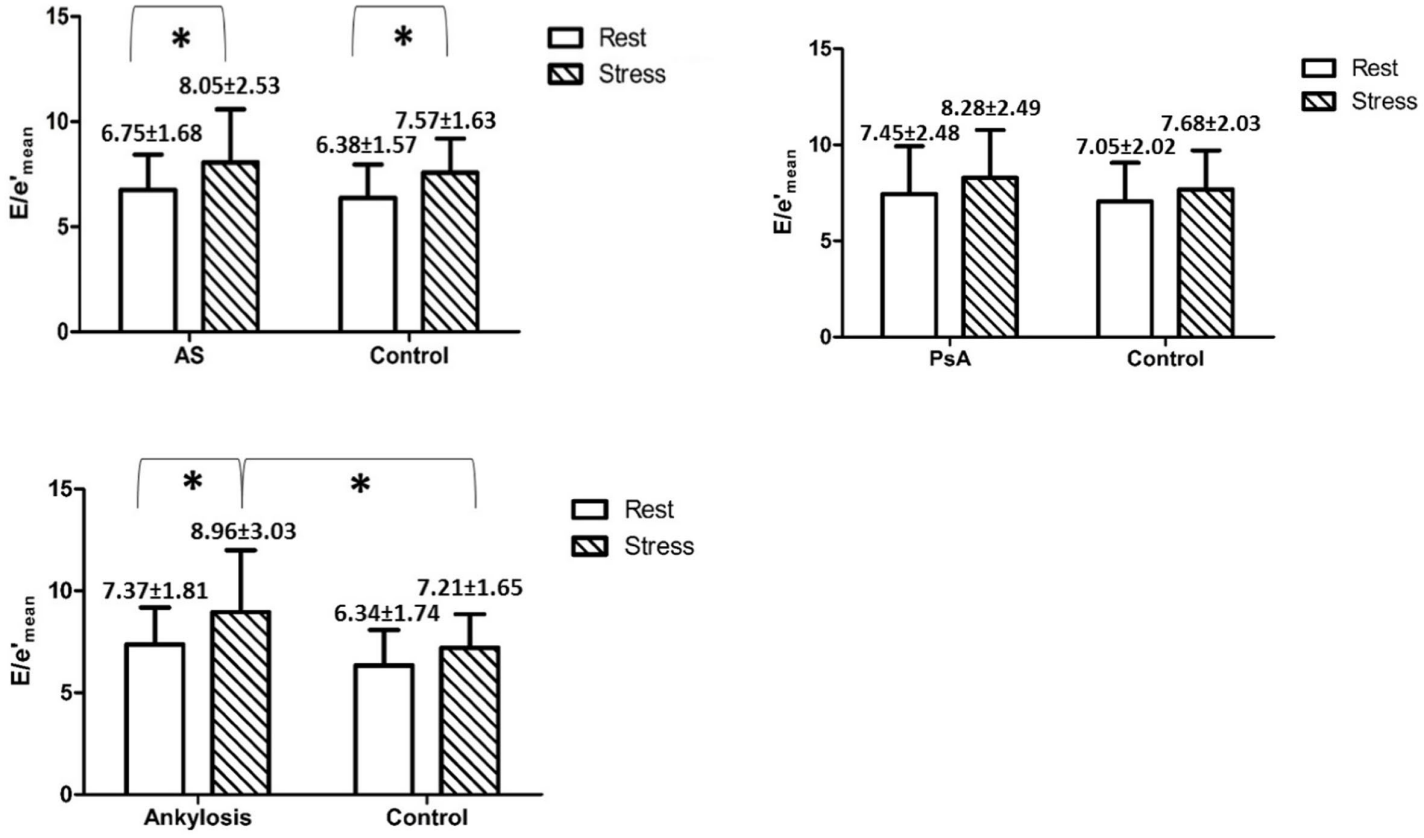
Shows mean *E*/*e*′ values with SD in and between the groups at rest and during peak stress. * indicates *p* < 0.05.

Regarding the estimation of the LV filling pressure at rest and under stress, we found that *E*/*e*′ significantly increased during stress in AS patients and in patients with ankylosis compared to rest. Moreover, at peak stress, *E*/*e*′ was significantly higher in patients with ankylosis than in controls (8.96 ± 3.03 vs. 7.21 ± 1.65, *p* < 0.05) (Figure 3 and Tables 1–3).

**Figure 4:**
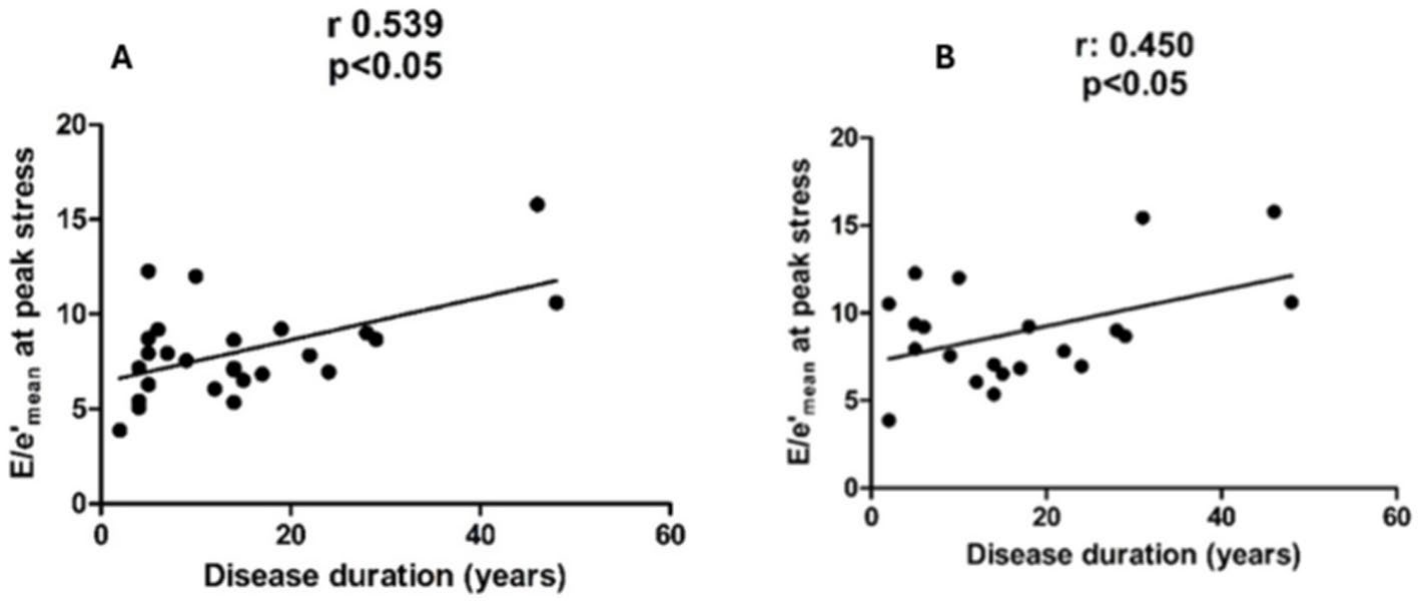
Shows a correlation between *E*/*e*′ at peak stress and disease duration in patients with AS (panel **A**) and in patients with ankylosis (panel **B**).

The disease duration was 13 (2–48) years in AS patients and 14 (2–48) years in ankylosis patients. This variable strongly correlated with *E*/*e*′ mean in patients with AS (panel A) and in patients with ankylosis (panel B), measured during peak stress (*r* = 0.539 and *r* = 0.45, *p* < 0.05), but not with TRV or PVR (Figure 4). After adjusting for age, disease duration remained significantly associated with *E*/*e*′ at peak stress in AS patients (*β* = 0.44 per year; 95% CI 0.01–0.17; *p* = 0.027), whereas this was no longer significant in ankylosis patients (*β* = 0.13 per year; 95% CI −0.09–0.16; *p* = 0.583).

## Discussion

To the best of our knowledge, this is the first comprehensive TTE study at rest and during stress using a semi-recumbent bicycle ergometer in AS and PsA patients. In addition to the standard cardiac parameters, the study further assessed valuable measures to evaluate the relationship between myocardial function, PVR, and the characteristics of AS and PsA. Our study included AS and PsA patients with axial involvement, with or without ankylosis. None of the patients had resting PH, moderate or severe valvular disease, or pulmonary fibrosis. All patients were also symptom-free at rest.

As a result of exercise, TRV and PVR were significantly higher in AS patients and patients in the ankylosing group compared to the controls. Generally, there is evidence that patients with axSpA have an impaired left ventricular longitudinal strain and an increased risk of diastolic dysfunction, with elevated left ventricular filling pressures measured by the *E*/*e*′ ratio, compared with the control group after adjusting for confounding factors (30). We also found that *E*/*e*′ showed a significant increase in AS patients during exercise and in the ankylosing group at peak stress compared to the control group. Diastolic dysfunction itself occurs more often in patients with a long-standing disease (31). In our study, left ventricular filling pressure elevation is strongly associated with disease duration in patients with AS and in patients ankylosis without any significant comorbidities. Close cardiological follow-up of AS and PsA patients with a long-standing disease may be particularly important, as prolonged illness alone can lead to left ventricular dysfunction.

Complex mechanisms can be assumed behind the increase in PASP and PVR caused by exercise. Chest wall rigidity and interstitial lung changes resulted in a restrictive ventilatory impairment, which can induce amplified hypoxic vasoconstriction during exercise, thereby increasing PVR and pulmonary pressure (32). In the study of O’Donnell et al. (33), the authors found that mechanical compromise with chest wall strapping in healthy individuals provoked severe dyspnea and exercise intolerance. Tidal volume responses were constrained in the face of increased ventilatory drive during physical activity. Altered breathing mechanics as a consequence of chest wall restriction also affect the filling of the left ventricle, which can be manifested as decreased cardiac output (34). However, our study did not find significant differences regarding cardiac output at rest and under stress in AS and PsA patients and controls. At the same time, the involvement of the myocardium, such as myocardial fibrosis and diastolic dysfunction, can contribute to the increase of *E*/*e*′ during stress (35). As a result of exercise, increased filling pressure is transmitted retrogradely to the pulmonary vasculature, resulting in increased PVR, pulmonary pressure, and reduced right ventricular contractility (36).

Exercise stress echocardiography has a relatively large literature in systemic sclerosis (SSc). PH resulting from pulmonary vascular remodeling is relatively common in SSc, developing in 8–12% of patients (37). The use of exercise stress echocardiography in SSc primarily aims at the early detection of pulmonary vascular remodeling, as the reduction in the reserve capacity of the vasculature under load (no recruitment phenomenon) is manifested in an abnormal increase in pulmonary pressure, compared to cardiac output, defined by stress-induced PH (38–40). Stress-induced PH in SSc may be a consequence not only of vascular remodeling, but also of pulmonary fibrosis and left ventricular diastolic dysfunction (40, 41). Therefore, it is essential to know the presence and extent of interstitial lung disease when establishing this indication. The prognostic role of stress-induced pulmonary hypertension is currently the subject of active research (42).

The restrictive breathing disorder resulting from the rigidity of the chest is widely investigated. In addition to the rigidity of the chest wall, in both AS and PsA patients, the PVR and PASP increase on exercise can also be caused by the subclinical fibrosis of the lungs. However, definitive fibrosis did not occur in our patient group; parenchymal lung abnormalities may impair the mechanisms of pulmonary vascular recruitment and distension that normally prevent the disproportionate increase in pulmonary pressure during exercise (43). According to Quismorio (44) study, lung involvement in patients with AS is approximately 16%, and in the majority of cases, lung involvement is of a restrictive pattern. Moreover, lung involvement in these patients may be asymptomatic.

The altered cardiac parameters examined in our study were associated with the underlying rheumatological diseases, including AS and PsA, particularly in patients with long-standing diseases and ankylosis. Alterations in the examined cardiac parameters can also indirectly draw attention to a potential restrictive lung process.

Cardiac magnetic resonance (CMR) has an established role with high sensitivity and specificity for the diagnosis of suspected myocardial involvement in PsA and AS patients. In AS and PsA patients, inflammatory processes in the myocardium can be detected even when clinical and laboratory parameters or routine echocardiography are normal (45). In a retrospective study, 50% of PsA patients showed late gadolinium enhancement on CMR (46).

In comparison with CMR and TTE, CMR is more sensitive for detecting active inflammation (edema) or fibrotic changes (late enhancement) in the myocardium, and its findings are consistent with myocardial biopsies (47). Speckle tracking-based strains can be used as an echocardiographic method to detect myocardial involvement in PsA patients. In a previous study using speckle tracking echocardiography, LV longitudinal strain was found to be similarly impaired in PsA patients compared to normal control subjects (−16.5% vs. −21.9), suggesting subclinical myocardial dysfunction (48). CMR can be calculated with hemodynamic measurements, such as volume, stroke volume, and cardiac output, and even exercise stress CMR is available. The advantages of echocardiography over CMR include cost-effectiveness, shorter duration, less discomfort, easy availability, and frequent repeatability.

### Limitations

Although the number of investigated patients is limited, the strength of our study is the homogeneity of the patient population without significant comorbidities. The study is cross-sectional; therefore, it holds some limitations, such as prevalence bias, and is not optimal for rare diseases. Stress echocardiography has traditionally been used for the diagnosis and risk stratification of ischemic heart disease. Subsequently, beyond this indication, its application was used to assess myocardial diseases, valvular diseases, diastolic dysfunction, and right hemicardial-pulmonary circulation. A limitation of our study is that the parameters measured during exercise echocardiography, such as *E*/*e*′, PASP, and PVR, were measured non-invasively. Although several previous publications have shown that the non-invasive determination of these parameters closely correlates with measurements obtained during invasive examinations, invasive pressure measurements during exercise would cause discomfort and possibly expose patients to complications. Exercise echocardiography is a non-invasive, radiation-free, and safe examination procedure. Its use in AS and PsA patients may, in the future, form a new indication for detecting subclinical early right ventricular-pulmonary circulation hemodynamic abnormalities associated with chest rigidity, as well as left ventricular diastolic dysfunction in the two patient groups. For established indications, multicenter studies with larger patient populations will be required.

## Conclusion

Hemodynamic changes during stress may unmask potential early pathophysiological processes in inflammatory rheumatic diseases, such as left ventricular diastolic dysfunction, the effects of chest wall restriction, and interstitial lung alterations. Therefore, exercise stress echocardiography might have a potential role as a non-invasive cardiologic investigation procedure in AS and PsA disease.

## Data Availability

The raw data supporting the conclusions of this article will be made available by the authors, without undue reservation.

## Glossary

PsA: Psoriatic arthritis
AS: Ankylosing spondylitis
TRV: Tricuspid regurgitation velocity
PVR: Pulmonary vascular resistance
PASP: Pulmonary arterial systolic pressure
LVEF: Left ventricular ejection fraction
LAVI: Left atrial volume index
TAPSE: Tricuspid annular peak systolic excursion
LVOT-VTI: Velocity-time integral of the left ventricular outflow tract
RVOT-VTI: Velocity-time integral of the right ventricular outflow tract
WU: Wood unit
RV: Right ventricle
BASDAI: Bath Ankylosing Spondylitis Disease Activity Index
DAS28: Disease Activity Score 28
eGFR: Estimated glomerular filtration rate
EACVI: European Association of Cardiovascular Imaging
PH: Pulmonary hypertension
CVD: Cardiovascular disease
MI: Myocardial infarction
SSc: Systemic sclerosis
TTE: Transthoracic echocardiography
CMR: Cardiac magnetic imaging
DMARDs: Disease-modifying antirheumatic drugs
ECG: Electrocardiography
